# Lanthanum Fluorogermanate Glass for Telecom Nonlinear Photonics: fs-Laser-Written Waveguides

**DOI:** 10.1021/acsomega.6c04632

**Published:** 2026-09-26

**Authors:** Renan Cunha, Kelly Tasso de Paula, Marcelo Barbosa de Andrade, Raphael Rodrigues Faleiros, Ana Flávia Teófilo da Silva, Fabia Castro Cassanjes, Gael Yves Poirier, Lino Misoguti, Cleber R. Mendonça

**Affiliations:** † São Carlos Institute of Physics, University of São Paulo, São Carlos 13566-590, Brazil; ‡ Departamento de Física, Instituto Tecnológico de Aeronáutica, São José dos Campos 12228-900, Brazil; § Physics Department, Federal University of Ouro Preto, Ouro Preto 35400-000, Brazil; ∥ Science and Technology Institute, 74347 Federal University of Alfenas, Poços de Caldas 37715-400, Brazil

## Abstract

Several rare-earth-based photonic devices rely on materials that combine wide transmission windows, favorable nonlinear properties, high ion solubility, and compatibility with waveguide inscriptions. While silica dominates current technologies, this work investigates a novel trivalent europium-doped lanthanum fluorogermanate glass. Such glasses stand out as promising candidates because of their optical transparency and thermal stability. Nevertheless, the suitability of LFG glasses for waveguide-based all-optical devices remains unclear, as optical parameters governing mode confinement, Kerr response, and laser damage threshold are still insufficiently characterized. Here, we investigate sub-bandgap femtosecond-laser processing of fluorogermanate glass, determining surface micromachining parameters and inscribing waveguides within the bulk. Incubation effects under 515 and 1030 nm irradiation are studied, and differences are discussed in terms of the ionization mechanisms. Also, photoluminescence spectroscopy indicates an increase in the local centrosymmetry around the Eu^3+^ sites following laser irradiation. Finally, we determine optical parameters for evaluating the performance of the material: the refractive index spectrum and group-velocity dispersion indicate a low-dispersion character of the glass. The nonlinear refractive index is determined via nonlinear ellipse rotation, reaching values up to three times higher than those of silica in telecommunication bands. Together with the three-photon figure of merit, our findings highlight the potential of fluorogermanate glass for waveguide-based all-optical devices for telecom photonics.

## Graphical abstract

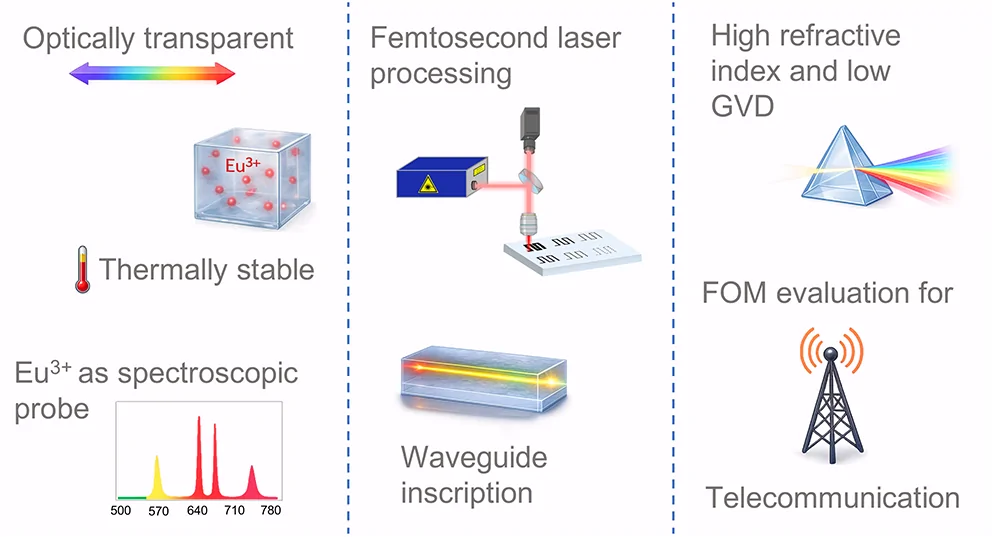

## 1 Introduction

Nonlinear optical technologies rely on a diverse set of material platforms, spanning organic chromophores and polymers,^1–6^ crystalline media,^7–13^ and a wide range of inorganic glasses.^14–20^ Organic materials often exhibit large optical nonlinearities and fast responses,^6,21–23^ while inorganic platforms generally offer superior thermal stability, chemical robustness, and long-term photostability under high optical intensities.^10,13,24–29^ In this landscape, glassy systems stand out as versatile and scalable hosts, combining broad transparency windows with compositional flexibility, enabling the tailored incorporation of dopants and functional species for advanced photonic applications.^30–35^

Despite the widespread use of silicate glasses in photonic technologies, the inherent limitations of silica, such as its relatively low nonlinear refractive index,^36,37^ have spurred the exploration of alternative glassy systems. Among these alternatives, germanate glasses have emerged as promising candidates due to their comparatively low phonon energy and enhanced solubility of rare-earth ions.^38–40^ In particular, the broader transmission window in fluorogermanate glasses makes them attractive materials for infrared integrated photonics, for which waveguide-based components such as directional couplers and optical switches are key building blocks for the next generation of photonic devices operating in telecommunication (telecom) bands.^41,42^ The incorporation of trivalent rare-earth (RE^3+^) ions further expands the potential of fluorogermanate glasses: the resulting stable, spectrally well-defined photoluminescence (PL) can be integrated with light-guiding structures for on-chip sensing, waveguide lasers, and optical amplifiers.^43–45^ This framework motivates the investigation of structural modifications and deterministic waveguide inscription in these glasses using potentially scalable fabrication strategies, while also assessing the optical response of RE^3+^-doped fluorogermanate compositions across the 1000–1600 nm range, which encompasses the O- (1260–1360 nm), E- (1360–1460 nm), S- (1460–1530 nm), C- (1530–1565 nm), and L-bands (1565–1625 nm) of optical communications.

Femtosecond-laser (fs) processing is particularly well-suited for this purpose, offering a versatile approach to induce localized modifications in transparent materials.^46–49^ It enables the fabrication of three-dimensional photonic structures, such as waveguides and gratings, with high spatial resolution and minimal thermal damage.^31,50–53^ Despite this potential, there remains a paucity of studies addressing the interplay between laser parameters and the resulting structural changes in RE^3+^-doped fluorogermanate glasses. Moreover, assessing these laser-induced changes is often challenging in doped glasses because their strong PL can obscure conventional structural signatures. However, doping with trivalent europium (Eu^3+^) provides an additional route to monitor structural modifications: the sharp f-f transitions of Eu^3+^ ions act as a sensitive spectroscopic probe of local symmetry changes.^54–56^ This makes Eu^3+^-doped fluorogermanate glass an appealing platform for investigating femtosecond laser-induced modifications and the optical properties of the host glass within telecom bands.

Therefore, this study investigates sub-bandgap fs-laser surface micromachining in a novel Eu^3+^-doped lanthanum fluorogermanate (LFG) glass, elucidating the associated laser-induced structural modifications, demonstrating guided-mode propagation in fs-laser-inscribed waveguides, and discussing the suitability of the host material for all-optical telecom-band applications. Here, we report on the surface and subsurface response of LFG glass to fs-laser irradiation with particular attention to the accumulation of laser-induced modifications under different irradiation conditions. Trivalent europium dipole transitions identified in the PL spectrum serve as spectroscopic probes to assess local symmetry changes in the glass following irradiation. In addition, we investigate femtosecond-laser-induced type I refractive index changes within the glass for waveguide inscription and investigate the coupling of a continuous-wave (CW) laser into the inscribed waveguides. Lastly, we estimate the group-velocity dispersion, nonlinear refractive index, and three-photon figure of merit to evaluate the suitability of the host glass for waveguide-based all-optical applications in the telecom bands.

## 2 Experimental Section

In this work, the Eu^3+^-doped LFG glass sample was synthesized by melt-quenching from a starting molar composition of 54.9GeO_2_–30LaF_3_–15K_2_O–0.1Eu_2_O_3_. Commercial starting powders GeO_2_ (99.99% from Acros) and K_2_CO_3_ (99.99% from Aldrich) were employed. Lanthanum fluoride LaF_3_ was synthesized using commercial lanthanum oxide La_2_O_3_ (99.9% from Acros) and sodium fluoride NaF (99% from Aldrich). La_2_O_3_ was dissolved in concentrated nitric acid, HNO_3_, at 80 °C, and sodium fluoride, NaF (99.99% from Aldrich), was dissolved in deionized water at room temperature. Both solutions were mixed in controlled volumes, resulting in a final molar ratio F/La around 3. The white precipitate was vacuum filtered, washed with deionized water, and dried at 100 °C for 24 h. X-ray diffraction measurements performed on the final white powder confirmed lanthanum fluoride LaF_3_ (JCPDS 32-483). Starting powders of GeO_2_, K_2_CO_3_ or Na_2_CO_3_, and LaF_3_ were weighed, ground in an agate mortar, heated in a covered platinum crucible at 900 °C for 1 h for carbonates decomposition, and melted in the same crucible at 1200 °C for 30 min. Then, the crucible was removed from the furnace for glass formation and annealed 20 °C below the glass transition temperature for 4 h before slow cooling back to room temperature. After this synthesis step, the sample was removed from the crucible, polished, and cut for further analyses.

We collected PL spectra using the Horiba LabRAM HR Evolution confocal mapping system using a 50×/0.9 NA objective lens and excitation line at 532 nm. We also obtained the absorption spectrum of the sample using a Shimadzu UV–vis 1800 spectrophotometer, which covers 190–1100 nm. The linear refractive index extracted from the Fresnel reflection contribution to the absorption data allowed us to estimate the group-velocity dispersion.

In the processing experiments, we modified the glass surface to probe its intrinsic response and optimize fabrication parameters, and we also induced a refractive index within the bulk to inscribe optical waveguides. To this end, we used a diode-pumped Yb:KGW laser system (CARBIDE, Light Conversion) equipped with a built-in pulse picker for on-demand pulse control, as well as tunable pulse duration, repetition rate, and wavelength. In both experiments, we employed excitation wavelengths of 515 and 1030 nm, with a pulse duration of 216 fs and a repetition rate of 197.5 kHz. We positioned the sample on a computer-controlled three-dimensional translation stage to ensure precise and constant-speed movement. A 40×/0.65 NA objective lens focuses the laser beam onto the surface of the material or into its volume, which is controlled by the z-stage. The energy and beam waist were determined experimentally. The microstructuring process was monitored in real time using a CCD camera aligned to the objective lens.

We evaluated the morphology and elemental mapping of the sample using scanning electron microscopy (SEM) combined with energy-dispersive X-ray spectroscopy (EDS) with a Hitachi TM-3000 tabletop microscope operating at 5 kV at a working distance of 4.1 μm and a Quantax EDX-Bruker system operating at 15 kV for elemental analysis.

The nonlinear refractive index of the sample was determined using the phase-sensitive nonlinear ellipse rotation (NER) technique.^57,58^ This Kerr-type nonlinear optical effect occurs when an intense elliptically polarized beam undergoes rotation of its major axis, with the rotation angle directly related to the third-order susceptibility of the material and, consequently, to the nonlinear refractive index *n*_2_. Here, an amplified Yb:KGW laser (Pharos PH1, Light Conversion) acts as a pump for an optical parametric amplifier (Orpheus, Light Conversion), which was tuned to 1000–1500 nm with a 150–180 fs pulse duration.

Figure 1 illustrates a linear polarizer and a quarter-wave plate that prepare the incident beam for an elliptical polarization, followed by two 3× objective lenses that focus and collimate the beam as it interacts with the sample mounted on a translation stage. A rotating polarizer at the detection stage modulates the signal, producing a squared cosine-like waveform as described by Malus’ law. Since the NER effect is significant only at the focal region, the waveform at the focus is phase-shifted relative to the waveform far away from the focus. The rotation angle is extracted from the phase shift with a photodiode connected to a dual-phase lock-in amplifier (LIA), which determines the ratio between the in-phase and quadrature components of the modulated signal.

**Figure 1:**
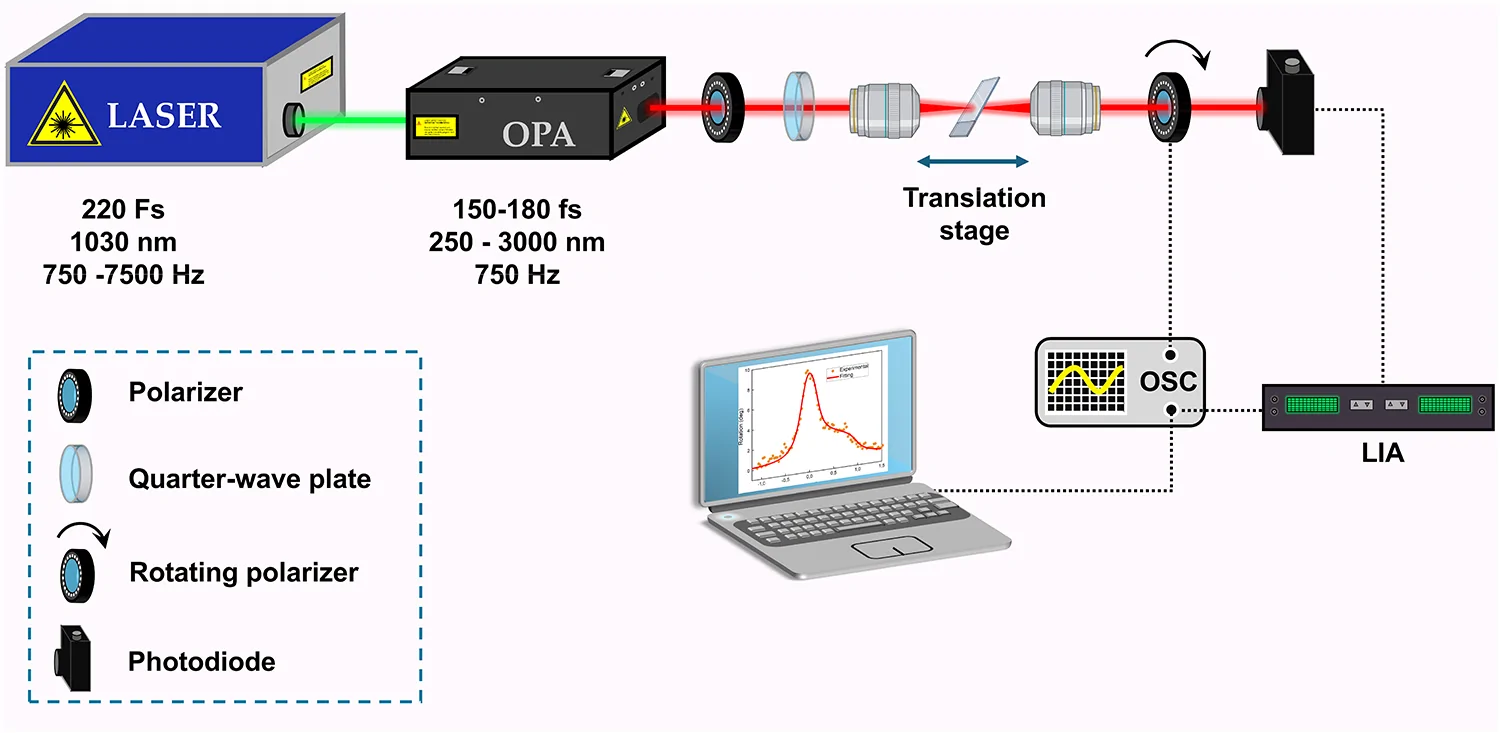
Phase-sensitive NER technique. The detected signal is monitored on the oscilloscope (OSC), and the ellipse rotation angle is extracted with a dual-phase lock-in amplifier (LIA) triggered by the rotating polarizer.

## 3 Results and Discussion

### 3.1 Absorption Spectroscopy

Figure 2 displays the UV–vis absorption spectrum obtained at normal incidence for the LFG glass in the 190–1100 nm range.

**Figure 2:**
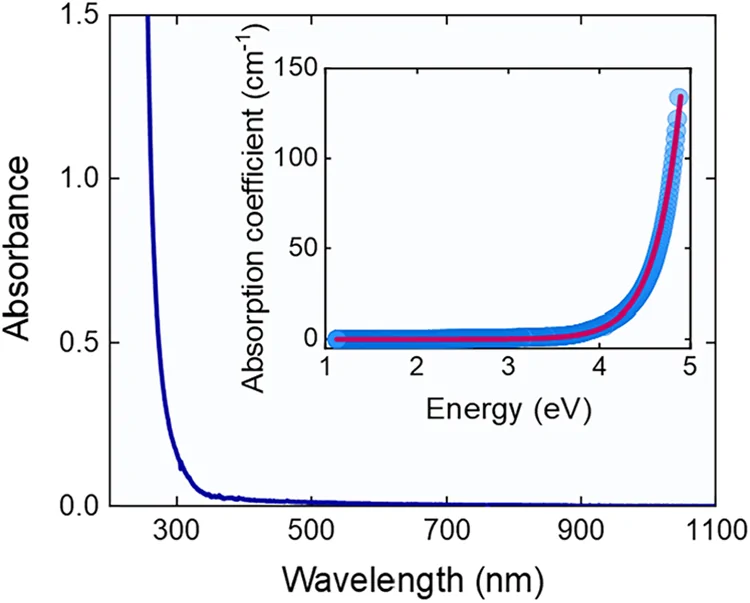
Linear absorption. Absorbance as a function of the wavelength (symbols, 190–1100 nm). The inset displays the linear absorption coefficient as a function of photon energy with a fit (line) for the bandgap and Urbach energy.

As shown, for wavelengths below 300 nm the absorption exhibits an abrupt increase. For longer wavelengths, the absorption decreases smoothly until about 500 nm, beyond which the FGL glass remains transparent. The inset of Figure 2 shows the spectrum of the linear absorption coefficient. The fit (line) to the data was performed using α = α_0_ exp­[(*E* – *E*_g_)/*E*_u_], where α_0_ is a constant, from which the optical bandgap energy of (4.8 ± 0.1) eV was calculated.^59,60^ The Urbach energy *E*_u_ = (2.82 ± 0.01) × 10^–1^ eV governs the transition between the absorptive and transparent regions of the absorption spectrum. A spectrum with *E*_u_ = 0 would exhibit an abrupt, perfectly defined vertical absorption edge. When *E*_u_ > 0, the absorption exhibits exponential decay rather than a sharp drop, a behavior known as the Urbach tail, as shown in the inset of Figure 2. This optical absorption characterization shows that transitions at wavelengths above 500 nm can only occur through strictly nonlinear processes. This ensures that the micromachining presented below at 515 and 1030 nm occurs in a regime free from linear absorption influences.

### 3.2 Micromachining

We investigated the effects of fs-laser irradiation on the surface of the LFG glass to determine laser operating parameters that optimize the micromachining process. We subjected the material to sub-bandgap excitation (2.41 eV, 515 nm; 1.2 eV, 1030 nm) at different fluences and number of fs-laser pulses, producing patterns on the surface and subsurface of the material, as displayed in Figure 3A. The number of overlapping pulses *N*_p_ interacting with the LFG glass is approximated by^61,62^

1 $$N_{p}\approx \frac{1.25fw_{0}}{v}$$

**Figure 3:**
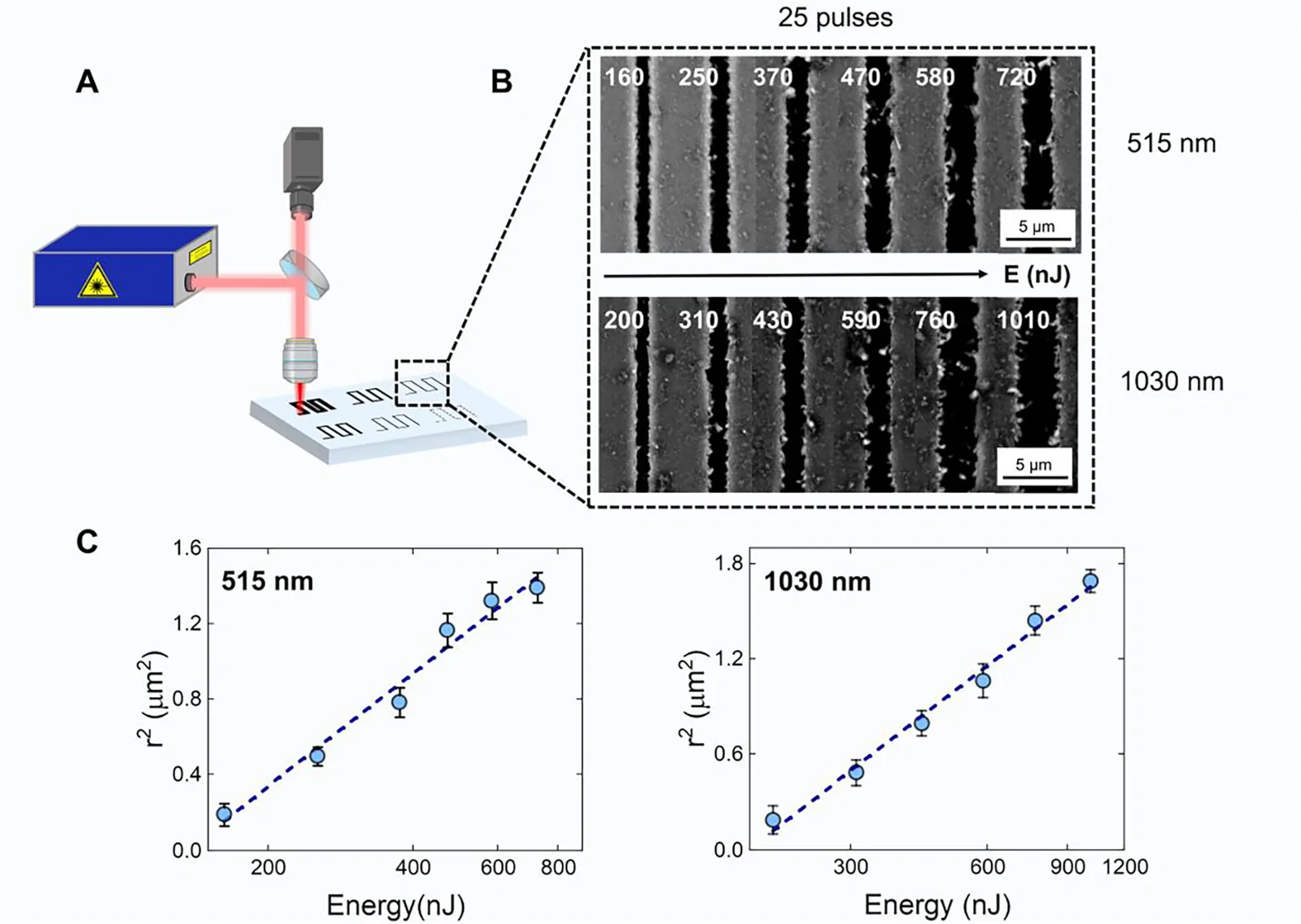
Surface micromachining. (A) Schematic of surface processing with different fs-laser energies and numbers of pulses. (B) SEM images of lines fabricated with 25 overlapping pulses at various energies using 515 and 1030 nm excitation. (C) Squared radius of the fabricated lines as a function of pulse energy (symbols) and best fit of eq 2 to the data (dashed line).

where *f*, *w*_0_, and *v* are the laser repetition rate, the beam waist, and the translation speed of the sample, respectively.

We determined *w*_0_ using the zero-damage method,^63^ which provides an experimental estimate of the beam waist rather than relying on the nominal numerical aperture of the objective used. The technique consists of evaluating the damage radius *r* produced by irradiation at different pulse energies *E*. Figure 3B shows SEM images of the damage created by irradiation with 25 overlapping pulses at 515 and 1030 nm. The data points in Figure 3C correspond to experimental measurements extracted from the SEM images, and the blue dashed line represents the fit according to^63^

2 $$r^{2}=\frac{w_{0}^{2}}{2}\ln\left(\frac{E}{E_{\mathrm{th}}}\right)$$

in which *E*_th_ is the damage threshold energy. The fit of eq 2 to the experimental data yields a beam waist of (1.3 ± 0.1) μm and 25-pulses damage threshold energy values of (136 ± 14) nJ and (179 ± 18) nJ for 515 and 1030 nm, respectively. The plot of the different damage threshold fluence values, calculated from *E*_th_ obtained by the zero-damage method, shows the transition from few- to multipulse excitation, demonstrating the incubation effect in Figure 4.

**Figure 4:**
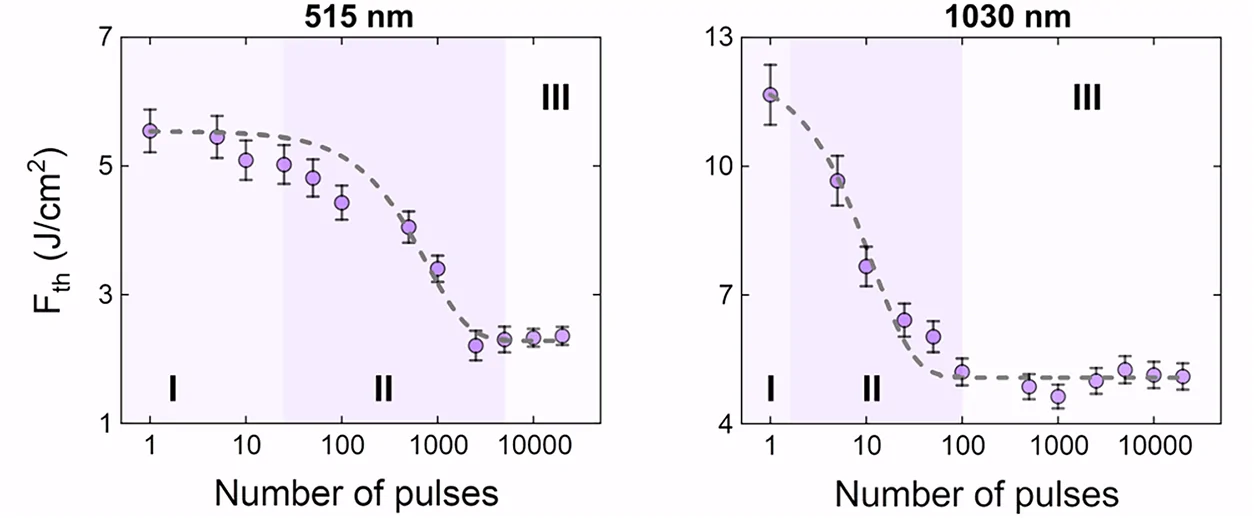
Incubation effect. Damage threshold fluence as a function of the number of overlapping pulses (symbols), with best fit of eq 3 to the data for 515 and 1030 nm excitation.

This effect is characterized by a continuous buildup of carrier and structural dynamics, facilitating the onset of damage. The overlap of pulses prevents the full relaxation of perturbations induced by each pulse, leading to subsequent pulses to interact with an increasingly perturbed lattice. Therefore, the general behavior of threshold fluence can be accounted for assuming that the relative reduction in fluence *F*_th_(*N*_p_) – *F*_th_(*N*_p_ – 1) is proportional to the threshold fluence for *N*_p_ – 1 pulses *F*_th_(*N*_p_ – 1). This yields the empirical relation^64^

3 $$F_{\mathrm{th}}\left(N_{p}\right)=F_{\mathrm{th}}\left(\infty \right)+\left[F_{\mathrm{th}}\left(1\right)-F_{\mathrm{th}}\left(\infty \right)\right]e^{-k\left(N_{p}-1\right)}$$

in which *F*_th_ (1) and *F*_th_ (∞) are single- and infinite-pulse threshold fluences, and *k* is the dimensionless incubation parameter accounting for the strength of the cumulative perturbation process. Fitting eq 3 to the experimental data gives *F*_th_ (1), *F*_th_ (∞), and *k* as (5.5 ± 0.6) J/cm^2^, (2.3 ± 0.2) J/cm^2^, and (1.3 ± 0.2) × 10^–3^, respectively, for 515 nm excitation. For 1030 nm excitation, we found (12 ± 1) J/cm^2^, (5.1 ± 0.5) J/cm^2^, and (0.09 ± 0.01). This difference in the parameters is also visually evident in Figure 4, where the incubation curve exhibits distinct shapes depending on whether micromachining was performed at 515 or 1030 nm. It can also be observed that the incubation process at 515 nm shows a much smoother dependence compared to 1030 nm: while a 60% drop in threshold fluence is only reached after approximately 2500 pulses for the former, a comparable 55% drop occurs within just 100 pulses for the latter. One plausible factor contributing to this difference lies in the ionization mechanism. To explore this contribution, we can calculate the Keldysh parameter associated with the laser–material interaction for both wavelengths. This allows us to discuss the dominant ionization mechanism in each case. The Keldysh parameter γ_K_ is given by^65^

4 $$\gamma_{K}=\frac{\omega }{e}\sqrt{\frac{m_{e}\varepsilon_{0}cn_{0}E_{g}}{I}}$$

in which ω is the angular frequency of the excitation laser, *e* and *m*_e_ are the electron charge and mass, ε_0_ is the vacuum permittivity, *c* is the speed of light in vacuum, *n*_0_ is the LFG glass refractive index at 515 (1030) nm, *E*_g_ is the bandgap energy, and *I* is the peak intensity of the Gaussian beam. As indicated by eq 4, the higher the intensity and the longer the wavelength, the lower the Keldysh parameter. The lowest value of γ_K_ is associated with *F*_th_(1), from which we found peak intensities of (2.4 ± 0.2) × 10^17^ W/m^2^ and (5.1 ± 0.5) × 10^17^ W/m^2^, and γ_K_ values of (2.6 ± 0.3) and (0.9 ± 0.1) for 515 and 1030 nm, respectively. On the other hand, the highest γ_K_ is associated with *F*_th_(∞), yielding values of (3.9 ± 0.4) and (1.3 ± 0.1) for 515 and 1030 nm, respectively. The Keldysh parameter indicates a multiphoton process when γ_K_ is greater than 1.5 and an electron tunneling otherwise.^66^ Therefore, the γ_K_ values indicate that the differences in the dynamics of the incubation effect observed for irradiation at 515 and 1030 nm in Figure 4 may be related to distinct ionization mechanisms. The ionization at 515 nm is governed predominantly by two-photon absorption, whereas quantum tunneling becomes the dominant mechanism at 1030 nm. Thus, with 515 nm excitation, two-photon absorption leads to incremental defect accumulation over many pulses. In contrast, at 1030 nm, incubation decays steeply, with efficient modification occurring within only a few pulses as a result of the tunneling through the strong field-distorted potential.

Besides the ionization pathways discussed above, additional mechanisms may contribute to the cumulative modifications, such as laser-induced structural defects. For that reason, we also investigated the effects of irradiation on the surface of LFG glass using EDS and PL spectroscopy. Figure 5A shows elemental distribution maps obtained by EDS. The main morphological effect of irradiation is the removal of material from the glass surface, leaving an ablated region that appears as a dark line in the images. Photoluminescence spectroscopy of the ablated regions provides structural information on the effects of irradiation. Figure 5B shows the PL spectra from regions irradiated at 1030 nm, with two energy values (137 nJ and 810 nJ) and 20,000 overlapping pulses. We can observe the expected set of Eu^3+^ transitions ^5^D_0_→^7^F_J_, where ^5^D_0_ and ^7^F_J_ correspond to the excited and ground-state multiplets of the 4f^6^ configuration, with the ^5^D_0_→^7^F_2_ band (611 nm) appearing as the most intense feature.^54–56^ As shown, higher-energy irradiation is associated with a decrease in the ^5^D_0_→^7^F_2_ emission band. This behavior is representative and was consistently found across different energies and numbers of pulses, being present under irradiation at both 1030 and 515 nm. This suggests a PL quenching mechanism induced by the laser interaction. The luminescence of Eu^3+^ features two transitions that are widely used as references for the local environment of the ion. The ^5^D_0_→^7^F_2_ transition (emission at 611 nm, R), allowed by electric dipole selection rules only in noncentrosymmetric sites, is highly sensitive to the local site symmetry around the Eu^3+^ ions. In contrast, the ^5^D_0_→^7^F_1_ transition (emission at 591 nm, O), which is magnetic dipole allowed, is relatively insensitive to symmetry change.^54–56^ Thus, the intensity ratio (R/O) between these two transitions provides a qualitative estimate of local symmetry and serves as an indicator of the structural transformations induced by femtosecond-laser irradiation. For the region irradiated with 20,000 pulses and 137 nJ (810 nJ), shown in Figure 5B, the R/O ratio is 7.05 (5.63). This suggests that the laser induces an increase in local symmetry. We further analyzed the dependence of the PL response on the irradiation conditions through the Pearson correlation matrix shown in Figure 5C. For each irradiation wavelength, each experimental condition was represented by four variables: number of pulses, pulse energy, R/O ratio, and ^5^D_0_→^7^F_2_ intensity. The Pearson correlation coefficient between each pair of variables was calculated as *r*_*xy*_ = cov­(*x*,*y*)/σ_*x*_σ_*y*_, where *x* and *y* represent the sets of values of any two variables across the experimental conditions, cov­(*x*,*y*) is their covariance, and σ_*x*_ and σ_*y*_ are their respective standard deviations. The resulting pairwise coefficients constitute the correlation matrix of Figure 5C, with values ranging from −1 to 1, where the sign indicates the direction of the linear correlation and the magnitude indicates its strength. Among the parameters, irradiation energy shows the strongest correlation with the R/O ratio, which decreases with increasing energy. Therefore, the decrease in the R/O ratio is consistent with an energy-dependent increase in local centrosymmetry around the Eu^3+^ ions induced by the femtosecond laser. Although no crystallization signature was observed, laser-induced structural reorganization may involve partial crystallization, with centrosymmetric cubic KLaF_4_ as one possible phase.

**Figure 5:**
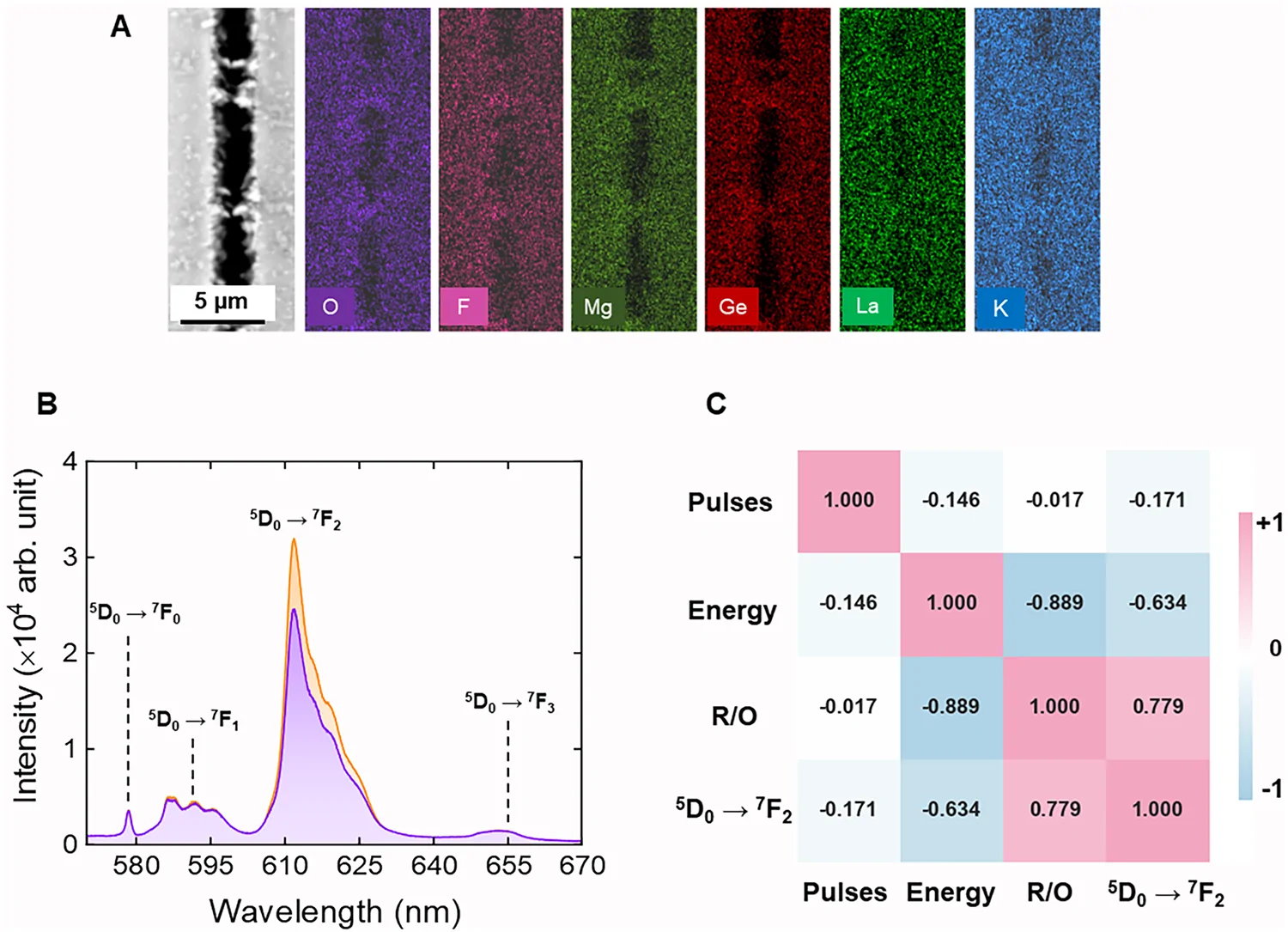
Structural transformations. (A) EDS mapping of a line fabricated with 25 overlapping pulses. (B) PL spectra of lines fabricated with 137 nJ (orange) and 810 nJ (purple) using 20,000 overlapping pulses. (C) Pearson correlation matrix of irradiation parameters and spectral signatures.

With the optimization of micromachining parameters, we investigated the inscription of optical waveguides in the LFG glass. In contrast to surface micromachining, no ablation occurs in this case. Instead, a selective increase in refractive index is induced within the glass volume.^67,68^ Figure 6A,B (Figure 6C,D) presents optical images of a fs-laser-inscribed waveguide and the cross-sectional view of its input facet, respectively, for 515 nm (1030 nm) excitation. The most homogeneous waveguides were obtained with low-energy irradiation. The energies used in the waveguides of Figure 6A,B are 190 nJ and 350 nJ, respectively. We observed the waveguide near-field profile using the objective lens-based coupling system shown in Figure 6E. A first objective lens (40×/0.65 NA) focuses a He–Ne laser (632.8 nm) at the input facet of the waveguides. The beam is then collimated and directed to a CCD camera by a second objective lens (40×/0.65 NA). Figure 6F (515 nm fabricated waveguide) and Figure 6G (1030 nm fabricated waveguide) displays the guided mode near-field profiles of the CW He–Ne laser beam, confirming single-mode guidance with approximately Gaussian intensity distributions, as evidenced by the line profiles.

**Figure 6:**
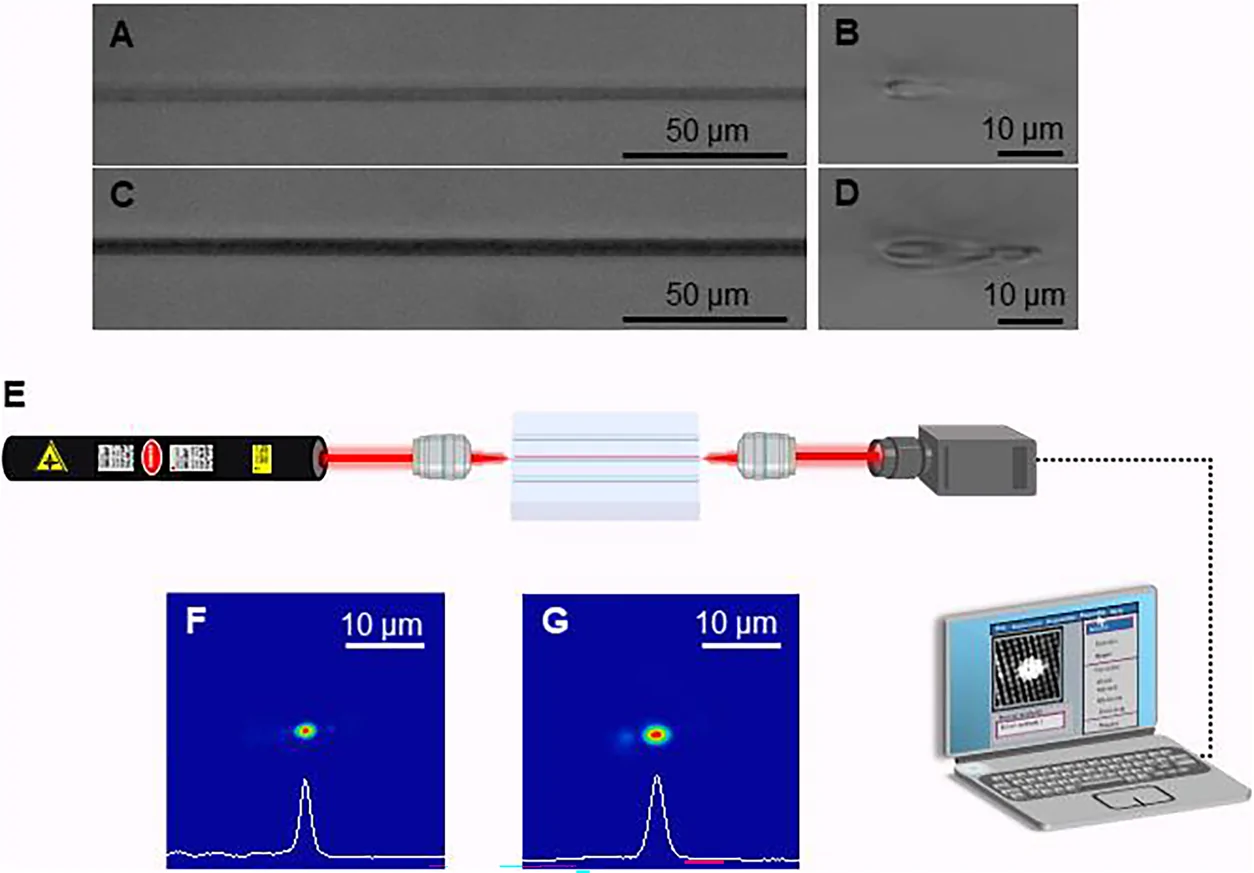
fs-laser inscription of optical waveguides. Longitudinal and cross-sectional views, respectively, of waveguides inscribed with fs-laser excitation at (A, B) 515 nm and (C, D) 1030 nm. (E) Coupling setup in which the guided-mode profile of a He–Ne laser is recorded on a CCD camera. Near-field intensity and corresponding line profile of the guided mode for waveguides fabricated at (F) 515 nm and (G) 1030 nm.

This indicates that the femtosecond-written LFG waveguides support well-defined optical modes, reinforcing the role of the host glass as a photonic platform. To further assess its suitability for operation in telecom bands, we determined the refractive and absorptive optical properties of the material.

The linear refractive index *n*_0_, group-velocity dispersion (GVD), and nonlinear refractive index *n*_2_ are optical quantifiers that account for phase-matching conditions, mode confinement, pulse broadening in ultrafast applications, self-phase modulation, and other intensity-dependent effects. In parallel, the linear and nonlinear absorption coefficients provide a measure of optical losses due to absorptive processes. Together, these parameters determine the suitability of the material for nonlinear photonic applications in the telecom window. Figure 7A shows the dispersion of the linear, intensity-independent refractive index extracted from the Fresnel reflection contribution in the absorbance spectrum of Figure 1. The open circles represent the experimental values of the index as a function of wavelength. The solid curve corresponds to the Sellmeier fit given by the expression

5 $$n_{0}=\sqrt{A+\frac{B_{1}\lambda^{2}}{\lambda^{2}-C_{1}}}$$

where *n*_0_ is the linear refractive index, λ is the wavelength, and *A*, *B*_1_, and *C*_1_ are the Sellmeier parameters. Fitting eq 5 to the data yields parameters *A* = 0.92147, *B*_1_ = 1.6324, and *C*_1_ = 0.0483 μm^2^. From the Sellmeier equation with the determined parameters, we relate the refractive index to the group velocity *v*_g_ through *d*(1/*v*_g_)*d*ω = *d* ^2^(*n*_0_ω/*c*)/*d*ω^2^. This allows us to estimate the GVD of LFG glass as a function of frequency, accounting for the temporal and spatial dispersion of the beam traveling through the material. Since this quantity is usually reported at 780 nm, we found a GVD of (422 ± 9) fs^2^/mm at 780 nm.

**Figure 7:**
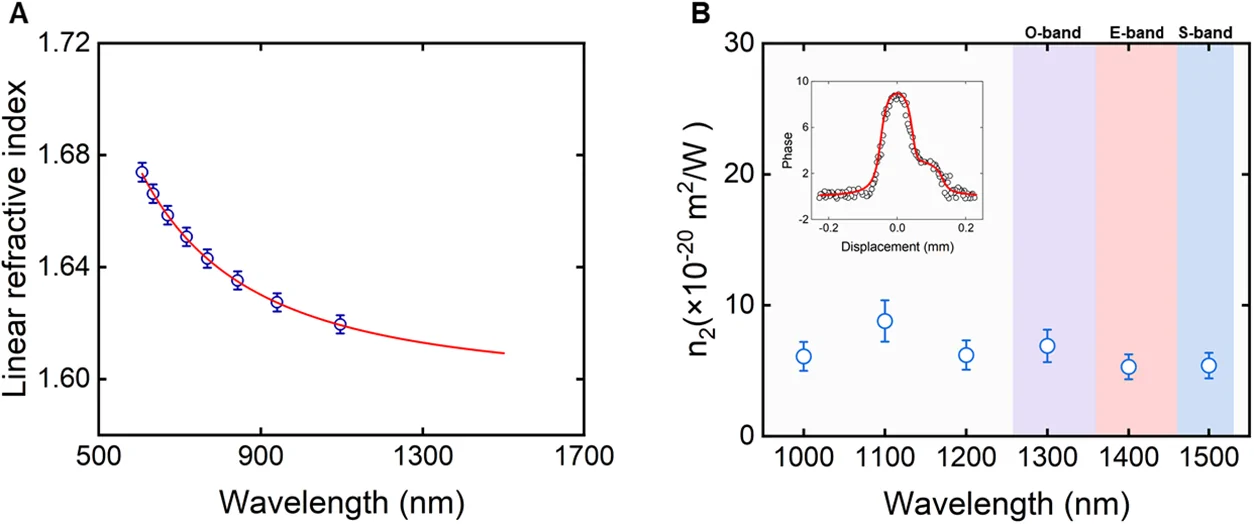
Refractive index dispersion. (A) Linear refractive index as a function of wavelength (symbols) with three-parameter Sellmeier fit (red line). (B) Nonlinear refractive index as a function of wavelength in the telecom bands. Inset: NER spectrum of the accumulated phase as a function of sample displacement relative to the laser focal distance.

To account for intensity-dependent refractive effects, we extracted *n*_2_ from measurements of the time-averaged NER angle α as the beam propagates through the LFG glass, which is given by^69^

6 $$\left\langle \alpha \left(z\right)\right\rangle =\frac{1}{\sqrt{2}}\frac{\omega }{c}\frac{\sin2\varphi }{2}\left[\frac{B}{4n_{0}^{2}\varepsilon_{0}c}\right]\left(n_{0}z_{0}\right)I\left[\tan^{-1}\frac{z_{b}}{z_{0}}-\tan^{-1}\frac{z_{a}}{z_{0}}\right]$$

where *z* is the position of the sample in the translation stage of the experimental setup in Figure 1, ω is the excitation angular frequency, *c* is the speed of light, φ is the angle between the incident beam polarization plane and the quarter-wave plate fast axis, ε_0_ is the vacuum permittivity, *z*_0_ is the Rayleigh length, *I* is the laser intensity, *z*_b_ = *z* + *L*/2*n*_0_ and *z*_a_ = *z* – *L*/2*n*_0_, where *L* is the sample thickness, and *B =* 8*n*_2_ *n*_0_^2^ε_0_ *c*/3. For comparison, we use silica as a reference material because of its widespread use in optical communications. Recent absolute NER measurements by our group yielded *n*_2_ values of approximately 2 × 10^–20^ m^2^/W at 1028 nm for silica.^70^ Figure 7B shows *n*_2_ values within telecom bands, where the LFG glass exhibits values about three times higher than silica. We found an average *n*_2_ of (7 ± 1) × 10^–20^ m^2^/W. The weak spectral variation of *n*_2_ over this range is consistent with the off-resonant character of the Kerr response, since the excitation photon energies are well below the 4.8 eV optical bandgap. Within the experimental uncertainty, the data show no significant dispersion of *n*_2_. However, *n*_2_ alone is insufficient to assess the suitability of a host material for photonic devices such as guided-wave directional couplers and optical switches in waveguides. Nonlinear absorption-induced loss mechanisms are also relevant for evaluating this suitability. Considering the absorption profile shown in Figure 2, the combined energy of three photons in the telecom range remains below the nominal 4.8 eV bandgap. However, the Urbach tail extends the absorption edge to lower energies, providing sub-bandgap states that can contribute to 3PA. Since the linear absorption in this region is already very weak, the corresponding 3PA contribution is also expected to be weak. For the purpose of this evaluation, the three-photon figure of merit is defined as FOM_3PA_ = *n*_2_/λα_3_ *I*, in which λ is the wavelength, α_3_ is the three-photon absorption coefficient, and *I* is the laser intensity.^71,72^ This figure of merit is a relevant quantifier that balances nonlinear refraction against potential nonlinear losses. In our measurements, no appreciable nonlinear absorption was observed. Therefore, rather than extracting α_3_ directly, we estimated an upper bound by taking the largest nonlinear transmittance variation compatible with the experimental uncertainty as the limiting 3PA signal.^73^ This limiting signal was then fitted with the analytical open-aperture Z-scan expression for 3PA reported by Corrêa et al.,^74^ using α_3_ as the fitting parameter. This procedure yielded an average upper-bound value of (2 ± 1) × 10^–23^ m^3^/W^2^ in the 1000–1500 nm range. Therefore, this yields a lower bound of 1.1 for FOM_3PA_. This FOM_3PA_ > 1 highlights the promising potential of LFG glass as a platform for next-generation nonlinear applications.

## 4 Conclusions

In this work, we evaluated the suitability of LFG glass for photonic applications in the telecom bands. Femtosecond-laser surface micromachining and direct waveguide inscription were investigated, followed by an assessment of the optical properties relevant for waveguide-based all-optical devices. Surface micromachining revealed two distinct incubation behaviors, which can be partly associated with different ionization mechanisms. The Keldysh parameter indicated that excitation at 515 nm is dominated by two-photon absorption, whereas processing at 1030 nm is governed by quantum tunneling. Femtosecond-laser irradiation also induced local structural modifications in the glass, as indicated by the PL spectra of the dopant ion, which suggested a laser-parameter-dependent increase in local centrosymmetry around the Eu^3+^ sites. Pearson correlation analysis further showed that this effect is strongly dependent on the ablation energy. Waveguides fabricated at both 515 and 1030 nm exhibited stable near-field single-mode profiles, demonstrating the ability of fs-laser writing to induce controlled refractive index changes in the LFG glass. This direct inscription route confirms the feasibility of producing three-dimensional guiding structures within the bulk, a key advantage for developing waveguide-based photonic devices without additional processing steps. To further benchmark the suitability of this platform for all-optical applications, we investigated the dispersive and nonlinear response of the host glass. The estimated GVD indicates relatively low dispersion across the telecom range, and the nonlinear refractive index reached approximately (7 ± 1) × 10^–20^ m^2^/W, while no appreciable three-photon absorption was observed. This result provides a lower bound of 1.1 for the three-photon figure of merit. Overall, the combination of a broad transparency window, robust fs-laser-written waveguides, and favorable nonlinear properties highlights LFG glass as a promising platform for integrated photonic devices operating in the telecom range.
